# Ulcerative colitis coexisting with hepatitis C: A rare occurrence

**DOI:** 10.1097/MD.0000000000036629

**Published:** 2023-12-15

**Authors:** Xiaoqiang Liu, Yisen Huan, Yubin Wang, Yingxuan Huang

**Affiliations:** a Department of Gastroenterology, First Hospital of Quanzhou Affiliated to Fujian Medical University, Quanzhou, Fujian, China.

**Keywords:** hepatitis C virus, sofosbuvir/velpatasvir, ulcerative colitis

## Abstract

**Introduction:**

We describe a rare case of a 54-year-old male diagnosed with both ulcerative colitis (UC) and hepatitis C virus (HCV), posing clinical challenges.

**Patient Concerns:**

The patient showed worsened UC symptoms, leading to further evaluations.

**Diagnosis:**

Dual diagnosis of UC and HCV was confirmed through endoscopy and serological tests, ruling out other hepatic causes. Interventions: Treatment involved methylprednisolone for UC and sofosbuvir/velpatasvir for HCV, with attention to drug interactions.

**Outcomes:**

Significant improvement was observed in both UC symptoms and HCV viral load post-treatment.

**Conclusion:**

This case underscores the need for nuanced treatment in managing concurrent UC and HCV, considering potential drug interactions and disease impacts.

## 1. Introduction

Ulcerative colitis (UC) and hepatitis C virus (HCV) are prevalent conditions with significant impact on patient health, yet they rarely occur simultaneously in 1 individual due to their distinct etiology and clinical presentation.^[1]^ Both are implicated in aberrant immune responses, albeit in different manners, contributing to their unique pathophysiology.^[2]^ This rarity of co-occurrence presents substantial challenges in terms of diagnosis and treatment. This article brings forth a unique case of UC complicated with HCV, with an aim to equip clinicians with knowledge to effectively identify and manage such rare instances. The goal is to facilitate timely diagnosis and effective therapeutic interventions, leading to improved patient outcomes. A statement regarding obtaining informed consent from the patient for the publication of detailed information in this case report should be included in the case report.

## 2. Case presentation

**Figure 1.**
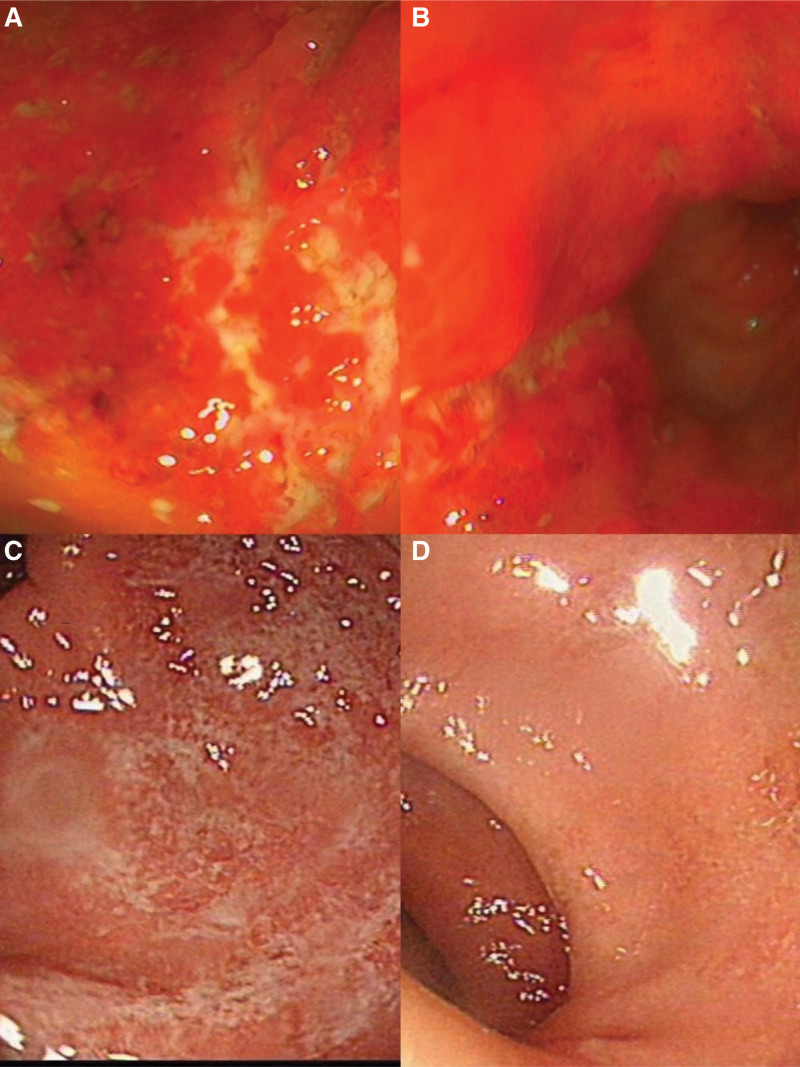
Changes in colonic mucosa before and after patient treatment. A and B represent the colonic mucosa condition before patient treatment. C and D represent the colonic mucosa condition after treatment.

A 54-year-old male patient presented with a 15-year history of recurrent mucopurulent bloody stool, which had worsened over the past 2 weeks. Fifteen years ago, the patient began experiencing an increased frequency of bowel movements, averaging 2 to 5 times per day. Stools were described as mushy, containing mucopurulent blood, and accompanied by tenesmus. He was diagnosed with UC following a colonoscopy at a local hospital. Initially, He was treated with Sulfasalazine orally, taking 1.0 gram (g) 3 times a day, for a year, then discontinued the medication independently. Although the frequency of mucopurulent bloody stools decreased during the medication period, symptoms recurred after cessation. Over the years, the patient sought treatment at various local hospitals. Seven years ago, he started taking mesalazine controlled-release tablets 1.0 g 3 times a day orally, but his adherence was inconsistent. During this period, the patient experienced intermittent recurrences of mucopurulent bloody stools. Five years ago, he was treated with oral steroids (specific drugs and doses unknown) at another hospital. He stopped steroid treatment after 1 year and independently discontinued the mesalazine controlled-release tablets 4 years ago. Since stopping the medication, the patient mucopurulent bloody stools have intermittently recurred. Half a month ago, the patient experienced another episode of mucopurulent bloody stools, with a frequency exceeding 20 times a day. The volume of mucopurulent bloody stool was larger than before, accompanied by occasional spasmodic pain in the lower abdomen, which improved after bowel movements. He self-treated with Yunnan Baiyao (a type of Chinese patent medicine), but the symptoms did not significantly improve. Consequently, he sought treatment at our hospital for further diagnosis and treatment. The outpatient department diagnosed him with UC and he was admitted to the hospital. He reported a weight loss of about 3 kg in the past 2 weeks. Physical examination upon admission revealed: Temperature 36.5°C, Pulse rate 80 beats per minute, Respiratory 19 beats per minute, blood pressure 126/88 millimeters of mercury (mm Hg), body mass index 31.2 kilograms per square meter (kg/m^2^). There was no rash on the skin all over his body, superficial lymph nodes were not palpably enlarged, his abdomen was soft, with slight tenderness in the lower left abdomen, no rebound pain, liver and spleen not palpable below the rib, bowel sounds were 4 times/min, no abnormalities in the anus and external genitalia, and limbs’ joints were normal. Auxiliary examination indicated: blood routine: white blood cells (WBC) 10.73 × 10^9/L, hemoglobin 148 g/l, platelet count 315 × 10^9/L; stool routine and occult blood: pus cells 4+/high power field (HP), WBC+/HP, red blood cells 4+/HP, occult blood positive; C-reactive protein: 73.72 milligram (mg)/L, erythrocyte sedimentation rate: 57 mm/h; stool calprotectin > 60 µg/g; antinuclear antibody 1:100 positive, atypical perinuclear anti-neutrophil cytoplasmic antibodies+++; biochemical: Albumin 37.6 g/L, aspartate aminotransferase 35 IU/L, alanine aminotransferase 45 IU/L; procalcitonin, d-dimer normal; interferon gamma release assays (−), purified protein derivative (−); Epstein-Barr virus DNA (−); cytomegalovirus (CMV)-IgM (−); Clostridium difficile antigen glutamate dehydrogenase and toxins A and B negative; carcinoembryonic antigen 6.09 ng/mL; lung computed tomography (−), venous Doppler ultrasound of both lower limbs (−); liver ultrasound real-time shear wave elastography: diffuse liver parenchymal disease, liver fibrosis classification: normal—mild. Gastroscopy indicated esophagitis, multiple polyps in the gastric fundus; colonoscopy: UC, modified Mayo score 11 points (colonoscopy description: Extensive congestion and edema of the mucosa were observed in the cecum, ascending colon, descending colon, sigmoid colon, and rectum, with the formation of erosion and ulcers, as shown in Fig. 1). Pathology report concluded chronic active inflammation with erosion and inflammatory exudation, granulation tissue proliferation, cryptitis, crypt abscess, glandular atrophy, reduced goblet cells, individual glandular dilation, irregular; special staining: acid-fast (-); immunohistochemistry: EB virus in situ hybridization: Epstein-Barr encoded region (-), CMV in situ hybridization: CMV(-).

**Figure 2.**
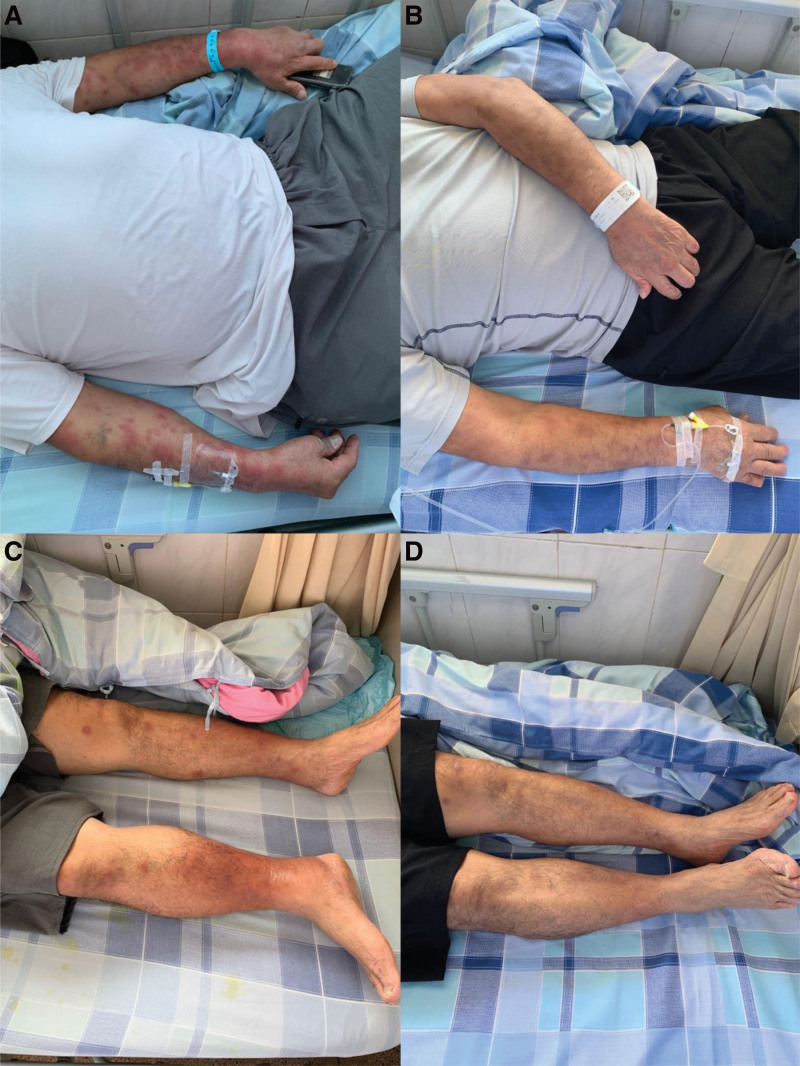
Changes in limb rash before and after patient treatment. A represents the rash condition on both upper limbs before patient treatment. B represents the rash condition on both upper limbs after treatment; C represents the rash condition on both lower limbs before patient treatment; D represents the rash condition on both lower limbs after treatment.

Upon reviewing the relevant test and examination results, we diagnosed the patient with “UC (Chronic Recurrent, E3, Active Phase, Severe).” On the first day of admission, the patient began treatment with mesalazine 1.0 g quater in die (qid) orally, mesalazine 4.0g quaque nocte enema, and gut microbiota regulation. He reported significant improvements in bowel movements and abdominal pain but continued to pass mucopurulent bloody stool approximately ten times a day. On the third day of hospitalization, the patient began to experience chills, shivering, and fever, with a maximum temperature of 38.3°C. A complete blood count revealed a WBC count of 12.53 × 10^9/L and a C-reactive protein level of 114.38 mg/L. A stool culture showed no Streptococcus (colonizing bacteria), suggesting a concurrent intestinal infection. As a result, the patient was administered moxifloxacin intravenously for antimicrobial therapy. On the fifth day of hospitalization, the patient frequency of passing mucopurulent bloody stool improved to 8 times per day. However, he continued to experience recurrent fever and developed nodular erythema on his limbs (Fig. 2). Further tests showed an HCV RNA level of 2.51E + 6 IU/mL, leading to a diagnosis of chronic hepatitis C. After consulting with the infectious diseases department, the patient was prescribed sofosbuvir/velpatasvir, 1 tablet quaque die (qd), for antiviral therapy. Given the severe UC diagnosis and the unsatisfactory results from the mesalazine treatment, we added methylprednisolone 40mg qd intravenously to his regimen, along with sofosbuvir/velpatasvir, 1 tablet qd orally, on the 11th day of hospitalization. On the 12th day of hospitalization, the patient stool consistency improved to a yellowish, semi-formed state, and he passed stool approximately 8 times per day with occasional mucopurulent bloody stool, but no abdominal pain or fever. The rash on his limbs also significantly improved, and the stool culture was negative. On the 16th day of hospitalization, the mode of methylprednisolone administration was changed from intravenous to oral at 40mg qd. On the 19th day of hospitalization, the patient stool became formed, was yellowish, and was passed 7 times a day, with no mucopurulent bloody stool. The rash on the limbs had almost completely disappeared (Fig. 2), and the patient was discharged. After discharge, the patient continued treatment with sofosbuvir/velpatasvir for chronic hepatitis C. After 12 weeks, a follow-up test for HCV RNA was negative. Post-discharge, the patient was gradually tapered off methylprednisolone over a period of 2 months and then switched to continued maintenance treatment with mesalazine. A year later, a follow-up colonoscopy.

## 3. Discussion

This case involves a patient with UC complicated by HCV. While both UC and HCV are common diseases, their simultaneous occurrence in a single patient is relatively rare. The etiology and pathophysiology of UC and HCV differ, but both are associated with immune responses, making their co-occurrence theoretically possible. The management of these diseases presents unique challenges due to their complex interaction. Immunosuppressive therapy for UC may increase the risk of HCV recurrence, while antiviral treatment for HCV may impact the course of UC. In this case, the patient was treated with methylprednisolone for UC and sofosbuvir/velpatasvir for HCV, both of which significantly improved the patient symptoms. This case underscores the importance of considering the potential impact of each disease and potential drug interactions when creating a treatment strategy for patients with UC and HCV. It also highlights the necessity of regular follow-ups to monitor disease progression and treatment response.

HCV is not an absolute contraindication to immunosuppressive therapy, but it may increase the risk of HCV reactivation, so close monitoring is required. There are currently no large-scale epidemiological data on HCV infection in inflammatory bowel disease (IBD) patients. A systematic review and meta-analysis examined the prevalence of hepatitis B virus and HCV infection in patients with IBD. The paper included 34 studies with a total of 17,022 IBD patients, and found that the prevalence of hepatitis B virus surface antigen in IBD patients was 3.3% and the prevalence of HCV antibody (anti-HCV) was 1.8%, which is not significantly different from the general population.^[3]^ The use of corticosteroids and immunosuppressants in IBD patients may affect the course of HCV. A study by Loras showed that corticosteroids may lead to a large replication of HCV and potential liver damage.^[4]^ Some scholars have reviewed 36 cases of drug-induced liver injury caused by infliximab, analyzed its clinical manifestations, biochemical indicators, autoimmune markers, and histopathological characteristics. The authors found that most patients showed hepatocellular injury and autoimmune phenomena, and about half of the patients needed to use corticosteroids due to elevated liver enzymes. The results showed that corticosteroid treatment can shorten the recovery time of liver injury, and there is no risk of recurrence after drug withdrawal.^[5]^ So far, no large-scale studies have assessed the impact of tumor necrosis factor-alpha (TNF-α) inhibitor treatment on the reactivation of HCV, but several small studies suggest a low risk. Generally speaking, as long as preventive treatment is used, treatment with TNF-α inhibitors will not significantly increase the risk of reactivation or reinfection of hepatitis C virus. It has been reported that even without specific anti-HCV treatment, the HCV load does not change significantly after 2 years of treatment with TNF-α inhibitors.^[6]^ In this case, we used prednisone for induction of remission treatment, and the patient did not have a large replication of HCV. Whether immunosuppressive therapy in IBD patients will affect the course of HCV is still inconclusive.

Whether the commonly used anti-HCV drug interferon will exacerbate IBD is uncertain, and the risk of anti-HCV treatment exacerbating IBD and drug interactions need to be fully considered. Direct-acting antiviral drugs (DAAs) are recommended for anti-HCV treatment. Before 2018, the main anti-HCV regimen in China was the PR regimen, pegylated interferon α (PEG IFN-α) combined with ribavirin treatment. This regimen is applicable to all genotypes of HCV in the absence of contraindications. In addition, a new type of drug, the DAAs against HCV, has been launched in Europe and the United States, and this class of drugs, such as sofosbuvir/velpatasvir, was launched in China in 2018. DAAs mainly exert antiviral effects by inhibiting some small molecular substances required in the maturation process of viral proteins, specifically including non-structural protein (NS)3/4A protease inhibitors, NS5A inhibitors, and NS5B polymerase inhibitors,^[7]^ for example, Sofosbuvir that has been launched abroad is a NS5B polymerase inhibitor. Specific treatment regimens include DAAs combined with ribavirin, different DAAs combined with each other, or PR combined with a DAAs. It is worth noting that the specific treatment regimen and course of DAAs are closely related to the genotype of HCV, so HCV genotype testing is required before medication.^[8]^ Some scholars have discussed the timing of treatment for IBD patients with chronic HCV infection, with a particular focus on the combined use of DAAs and biologics. The paper, based on a literature review and expert opinion, proposed 3 possible timing strategies: sequential strategy, first controlling the active phase of IBD with biologics, and then treating HCV infection with DAAs; simultaneous strategy, starting DAAs and biologics at the same time; reverse strategy, first clearing HCV with DAAs, and then treating IBD with biologics. The paper pointed out that these 3 strategies each have their own advantages and disadvantages, and the decision should be made based on the specific situation of the patient and clinical judgment. Recently published data on DAAs are also very encouraging in IBD patients. All strategies can be considered safe and effective.^[9]^ Therefore, in this case, we decided to use DDAs for anti-HCV treatment, and the patient final outcome was safe and effective.

In summary, for IBD patients, it should be noted that IFN-α can act as a pro-inflammatory factor to mediate Th1 type immune response, which may exacerbate UC,^[10]^ so the timing and pros and cons of IFN-α treatment need further study. In addition, the antiviral drug ribavirin can promote methylation by inhibiting guanosine monophosphate dehydrogenase, reducing the content of the active product 6-thioguanine nucleotides, which may increase the hepatotoxicity of purine drugs. Whether DAAs and immunosuppressants have interactions is not very clear, and their combined use needs to be cautious.

## Author contributions

**Conceptualization:** Xiaoqiang Liu.

**Project administration:** Yisen Huang.

**Supervision:** Yubin Wang.

**Writing – original draft:** Yingxuan Huang.
